# Brain size variation along altitudinal gradients in the Asiatic Toad (*Bufo gargarizans*)

**DOI:** 10.1002/ece3.7192

**Published:** 2021-01-26

**Authors:** Zhongyi Yao, Yin Qi, Bisong Yue, Jinzhong Fu

**Affiliations:** ^1^ Chengdu Institute of Biology Chinese Academy of Sciences Chengdu China; ^2^ College of Life Sciences Sichuan University Chengdu China; ^3^ University of Chinese Academy of Sciences Beijing China; ^4^ Department of Integrative Biology University of Guelph Guelph ON Canada

**Keywords:** altitudinal gradient, Asiatic toads, brain size, expensive brain, functional constraint

## Abstract

Size changes in brain and brain regions along altitudinal gradients provide insight into the trade‐off between energetic expenditure and cognitive capacity. We investigated the brain size variations of the Asiatic Toad (*Bufo gargarizans*) across altitudes from 700 m to 3,200 m. A total of 325 individuals from 11 sites and two transects were sampled. To reduce confounding factors, all sampling sites within each transect were within a maximum distance of 85 km and an altitudinal difference close to 2,000 m. Brains were dissected, and five regions were both measured directly and with 3D CT scan. There is a significant negative correlation between the relative whole‐brain volume (to snout‐vent length) and altitude. Furthermore, the relative volumes (to whole‐brain volume) of optic tectum and cerebellum also decrease along the altitudinal gradients, while the telencephalon increases its relative volume along the gradients. Therefore, our results are mostly consistent with the expensive brain hypothesis and the functional constraint hypothesis. We suggest that most current hypotheses are not mutually exclusive and data supporting one hypothesis are often partially consistent with others. More studies on mechanisms are needed to explain the brain size evolution in natural populations.

## Graphical abstract

We explored the size variation of brain and brain regions along altitudinal gradients. The relative size of overall brain, optic tectum, and cerebellum decreases with increasing altitudes, while the relative size of telencephalon increases with altitudes.

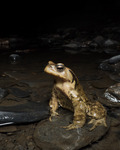

## 1 INTRODUCTION

Brain size variation plays an important role in animal adaptation processes (Healy & Rowe, 2007; Montgomery et al., 2016). In vertebrates, brain size is positively correlated with number of neurons and thus is often served as an indicator of brain functional capacity (Marhounová et al., 2019). Numerous studies have examined brain size variations across vertebrate groups, including fishes (Axelrod et al., 2018; Tsuboi et al., 2016; Yopak et al., 2010), amphibians (Amiel et al., 2011; Jiang et al., 2015), reptiles (Amiel et al., 2011; De Meester et al., 2019; Sampedro et al., 2008), birds (Rehkämper et al., 2008; Tsuboi et al., 2018), and mammals (LemaîTre et al., 2009; Powell et al., 2017; Tsuboi et al., 2018). Furthermore, many of these studies explored potential causes of brain size variation, including habitat, behavior, and social complexity (e.g., Axelrod et al., 2018; De Meester et al., 2019; Towe & Mann, 1995). Consequently, several key issues have been addressed and alternative hypotheses proposed; however, the results are often in conflict and controversies largely remain (De Meester et al., 2019; Healy & Rowe, 2007).

Which factors drive the evolution of brain size is one of these key issues. The expensive brain hypothesis (EBH) suggests that the evolution of brain size is constrained by either the total energetic input or the energy allocated to the brain, and predicts a correlation between the lowest level of steady energetic input and the brain size (Heldstab et al., 2016; Isler & van Schaik, 2009; Navarrete et al., 2011). Several studies demonstrated that energetic constraints imposed by environmental seasonality and hibernation play a crucial role in mammalian brain size evolution (Heldstab et al., 2018, 2019; van Woerden et al., 2012). A recent comparative analysis of anuran species also supported the EBH (Luo et al., 2017). The cognitive buffer hypothesis (CBH) postulates that a large brain can benefit an individual via enhanced behavioral flexibility to cope with novel or changing environments (Gu et al., 2017; Sol et al., 2008; van Woerden et al., 2012). CBH predicts a link between brain size and cognitive ability (Neubauer et al., 2018). Experimental evidence confirmed that brain size is associated with reversal‐learning (Buechel et al., 2018), behavior flexibility (Herczeg et al., 2019), and antipredator behavior (Kotrschal, Buechel, et al., 2015). Furthermore, habitat complexity is correlated with brain size or brain region sizes in sunfish (*Lepomis gibbosus*; Axelrod et al., 2018) and bats (Safi & Dechmann, 2005). Nevertheless, the habitat complexity does not affect the relative brain size of squamates, and solitary species have relatively larger brains than social species (De Meester et al., 2019). In an anuran species, *Fejervarya limnocharis*, neither CBH nor EBH is supported (Mai et al., 2017).

Whether different regions of the brain evolve in a concerted fashion or independently is another important issue. The developmental constraint hypothesis (DCH) proposes that different regions of the brain tend to evolve together as the selective pressures likely work on mechanisms that affect the growth of all components in a concerted way (Kotrschal et al., 2017; Montgomery et al., 2016). Experimental evidence from sunfish (*Lepomis gibbosus)* and guppies (*Poecilia reticulata*) supports the concerted model (Axelrod et al., 2018; Kotrschal et al., 2017). On the other hand, the functional constraint hypothesis (FCH) posits that selection acts on distributed functional systems which connect different subcomponents (Montgomery et al., 2016), and therefore, patterns of mosaic changes are at the level of functional systems (Kotrschal et al., 2017). Several studies on anuran species support FCH (Liao et al., 2015; Zeng et al., 2016).

A variety of approaches have been applied to brain size comparison, which may have contributed to the current lack of consensus. First, both relative brain size and absolute size have been used. Body size varies dramatically in vertebrates, as well as brain sizes (Kotrschal, Corral‐Lopez, et al., 2015; Powell et al., 2017). It is well established that brain size is positively correlated with body size both within and between species (Font et al., 2019; Tsuboi et al., 2018; Zeng et al., 2016). It has been questioned whether absolute or relative brain size is an adequate proxy of cognitive capacity; however, in regard to energetic trade‐off, relative brain size is a more suitable measurement (Kaas, 2016). Several studies took both absolute size and relative size into consideration (Burns et al., 2009; Mai et al., 2017), but the majority of recent studies focused on relative size (Gu et al., 2017; Kotrschal, Buechel, et al., 2015; Kotrschal et al., 2017). Both body length and body mass have been used as covariates (Burns et al., 2009; Garamszegi et al., 2005; Gu et al., 2017; LemaîTre et al., 2009). Second, both intraspecific and interspecific comparisons have been employed. The wide application of phylogenetic comparative methods in interspecific comparison has successfully generated hypotheses regarding the evolution of functional traits (Liao et al., 2016; Marhounová et al., 2019; Safi & Dechmann, 2005). Intraspecific variations, however, are more powerful in testing mechanistic hypotheses (Levis et al., 2017). Comparison within a species or between closely related species would exclude confounding ecological backgrounds associated with different species (Kotrschal, Corral‐Lopez, et al., 2015; LemaîTre et al., 2009) and allow us to focus on the brain variables (Axelrod et al., 2018; Gonda et al., 2013).

An ecological context is essential in intraspecific comparison. Altitudinal gradient is one of the most frequently used ecological discrepancies in revealing biodiversity and the evolutionary mechanisms behind it (Hodkinson, 2005; Keller et al., 2013; Navas, 2002). High altitude environments are characterized by hypoxia, low temperature, high UV radiation, and high climatic variability both daily and seasonally (Storz et al., 2010). Hypoxia is the most representative stress at high altitudes. The decrease in oxygen partial pressure is independent of latitude, season, diurnal rhythms, or meteorological conditions (Bouverot, 1985; Ivy & Scott, 2015; Storz et al., 2010). As a consequence, all organisms living in high altitudes have to overcome the reduced supply of oxygen. The function of the brain depends on the continuous supply of oxygen and glucose (Olesen, 1986; Singer, 2007), and when the supply of oxygen and glucose is interrupted, brain function will deteriorate (Singer, 2007). Therefore, high altitude represents strong directional selection (Tate et al., 2017), and trait variations among populations can be explained by the gradients (Albert et al., 2010). The altitudinal gradient system provides an explicit ecological context for examining brain size variation.

**FIGURE 1:**
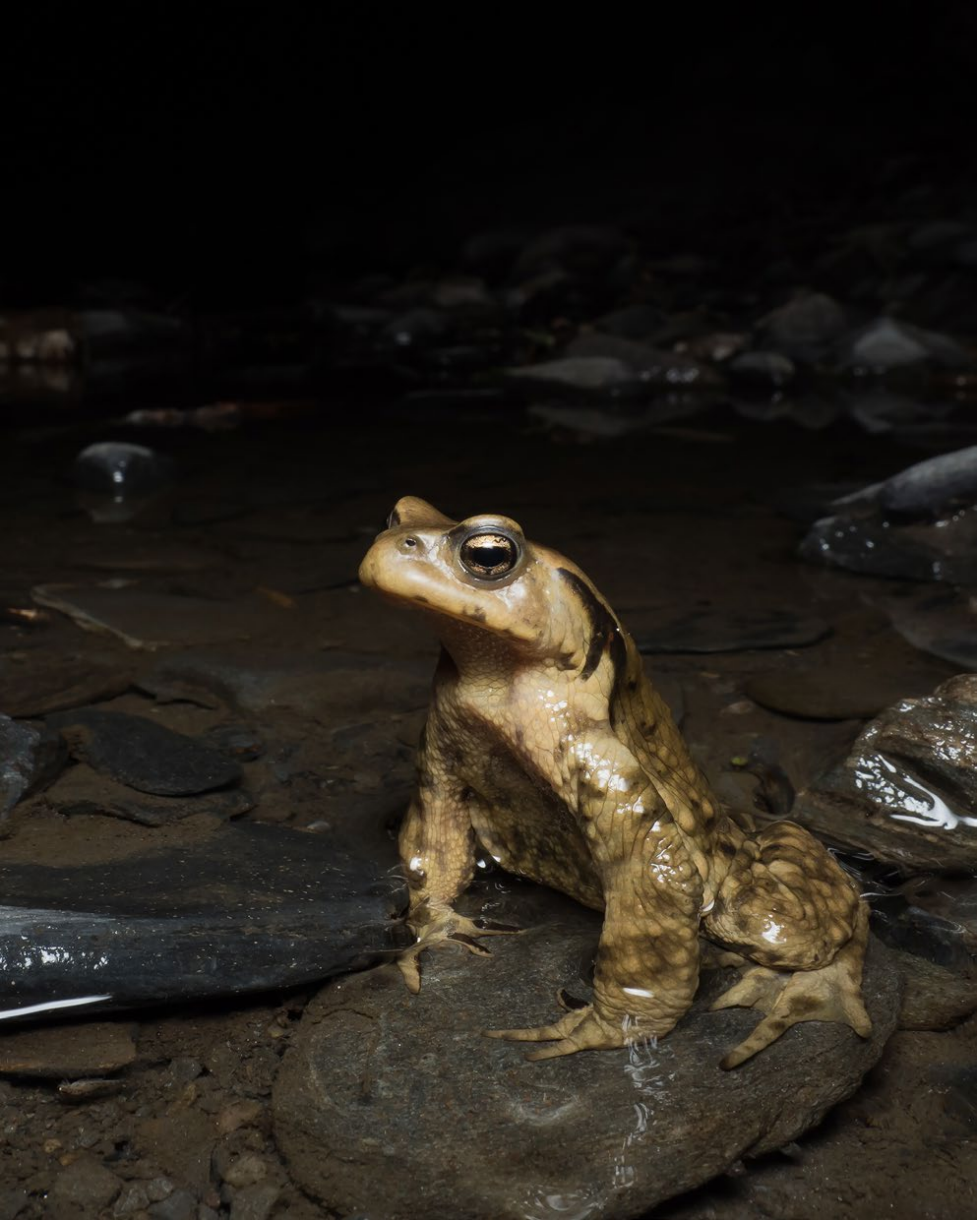
A male Asiatic toad at a breeding pond. Nuptial pad can be seen on its front toes

The Asiatic Toad (*Bufo gargarizans*, Figure 1) is widely distributed in eastern Asia and occurs in a variety of habitats, particularly an altitudinal range of 120 m to 4,300 m above sea level (AmphibiaChina, 2020). It is a common species, and high population density makes sampling and studying the species relatively easy. The toad populations of the Hengduan Mountains of western China are particularly suitable for an altitudinal gradient study. This region features sharp‐rising mountains and deep‐cut valleys, and within a short spatial distance, the altitudes often range from 400 m to above 3,000 m. As a result of this special landscape, the environmental variables change dramatically along the altitudinal gradient in a short spatial distance.

We examine the intraspecific size variation of brain and brain regions of the Asiatic Toad along altitudinal gradients, and more specially, we test four hypotheses related to brain size evolution using this system. The EBH predicts small brain size at high altitudes as hypoxia, short active time, and other stressors set energetic limitations to the toads. The CBH predicts that that the brain size of toads from high altitudes would be enlarged to enhance the cognitive ability or behavioral flexibility, and to overcome the high daily and seasonal environmental variability associated with high altitudes. Additionally, the DCH predicts that the brain regions would change size in a concerted manner, while the FCH suggests that different brain regions would vary differently depending on their functional importance. We predict that toads at high altitudes would have disproportionally larger telencephalon and smaller cerebellum compared with their lower‐altitude counterparts. Hypoxia at high altitudes severally limits energy supply during locomotion, and toads likely use more walking and less jumping, which would lead to relaxed control of posture and therefore, a smaller cerebellum. Meanwhile, dehydration and short breeding seasons at high altitudes would require toads to find a breeding pool in a shorter time; a better spatial learning capacity associated with a larger telencephalon is likely beneficial.

## 2 MATERIALS AND METHODS

### 2.1 Sampling

**FIGURE 2:**
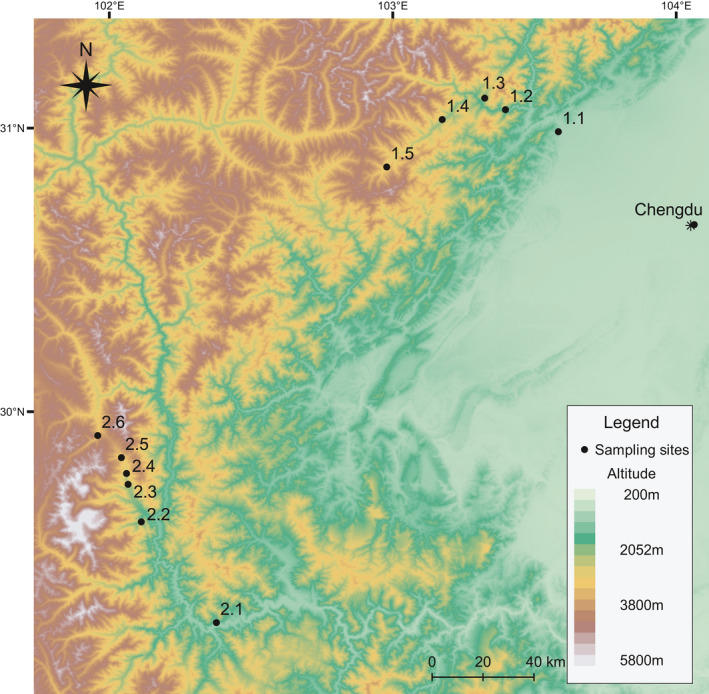
Map of southwestern China with sampling sites. Sites 1.1–1.5 are from transect 1, and sites 2.1–2.6 are from transect 2. The city of Chengdu (*) is marked as a reference point

A total of 325 individuals from 11 sites were collected during breeding seasons of 2018 and 2019 (Figure 2, Table 1). We sampled two transects. Transect 1 is located near the Wolong National Nature Reserve, and we sampled five sites within a distance of approximately 60 km. Transect 2 is located near the Mt. Gongga, and we sampled six sites within a distance of approximately 84 km. In general, we tried to sample sites that were altitudinally 300 m to 500 m apart along a valley when possible. To minimize potential geographic confounding factors, sites within the same transect were selected within the shortest distance possible. To avoid autocorrelation, the two transects were located at two different mountain ranges with the closest points more than 140 km apart (Figure 2). We sampled breeding populations to control potential effects of age and seasonality on brain morphology (Axelrod et al., 2018; Ebneter et al., 2016; Jiang et al., 2015; Puga et al., 2018). The breeding season of this species is at the very beginning of the active season; for populations at low altitudes, it lasts from the end of December to early February, while for high altitude populations, the season lasts from April to May. Thus, sampling times of different sites were in different months as the phenology was delayed along altitudinal gradients (Table 1). All animal procedures were carried out in accordance with the approved protocols from the Animal Care and Use Committee at the Chengdu Institute of Biology, Chinese Academy of Sciences (Permit number: 20180820).

**TABLE 1:** Information of the sampling transects and sampling sites

Transect	Site	Date	Altitude (m asl)	Longitude	Latitude	Specimens (♂:♀)
1	1.1	2018.2.6	785	103.5839	30.9873	20:18
	1.2	2018.2.28	1,223	103.3974	31.0650	20:17
	1.3	2018.3.2	1,780	103.3241	31.1058	22:20
	1.4	2018.3.23	2,106	103.1742	31.0302	29:10
	1.5	2018.4.5	2,759	102.9785	30.8630	15:15
2	2.1	2019.1.12	926	102.3777	29.2567	16:16
	2.2	2019.2.19	1689	102.1129	29.6119	12:8
	2.3	2019.2.20	2,136	102.0662	29.7442	13:16
	2.4	2019.4.6	2,452	102.0607	29.7825	11:11
	2.5	2019.4.9	3,015	102.0426	29.8382	5:1
	2.6	2019.4.9	3,239	101.9592	29.9161	15:15

### 2.2 Linear measurement‐based volume estimate (*V*_LM_)

All individuals were euthanized with 0.25% MS‐222 solution (Dodd, 2010; Mitchell et al., 2020). Specimens were fixated and stored in neutralized 10% formalin (Carson et al., 1973). After three months in storage, specimens were photographed in both dorsal and ventral views with an Olympus camera (Em5mark2). The coronal plane of all specimens was ensured to be parallel to the sensor of the camera, and the focal length was fixed. A ruler was placed in all photographs as a reference. Measurements were taken from the photos using ImageJ (v1.53d). The snout‐vent length (SVL) was measured to the nearest 0.001 mm, and the measurement was repeated three times for each individual.

Brains were dissected out, and the cranial nerves, pineal organ, and meninx were removed. The pituitary glands were also removed but the pituitary infundibulum was preserved (Duellman & Trueb, 1994; Kardong, 2012; Nieuwenhuys et al., 2014). Similarly, photographs of these brains were taken from dorsal, lateral, and ventral views. The coronal plane and sagittal plane were parallel to the camera sensor, and a ruler was placed in all photographs as a reference.

**FIGURE 3:**
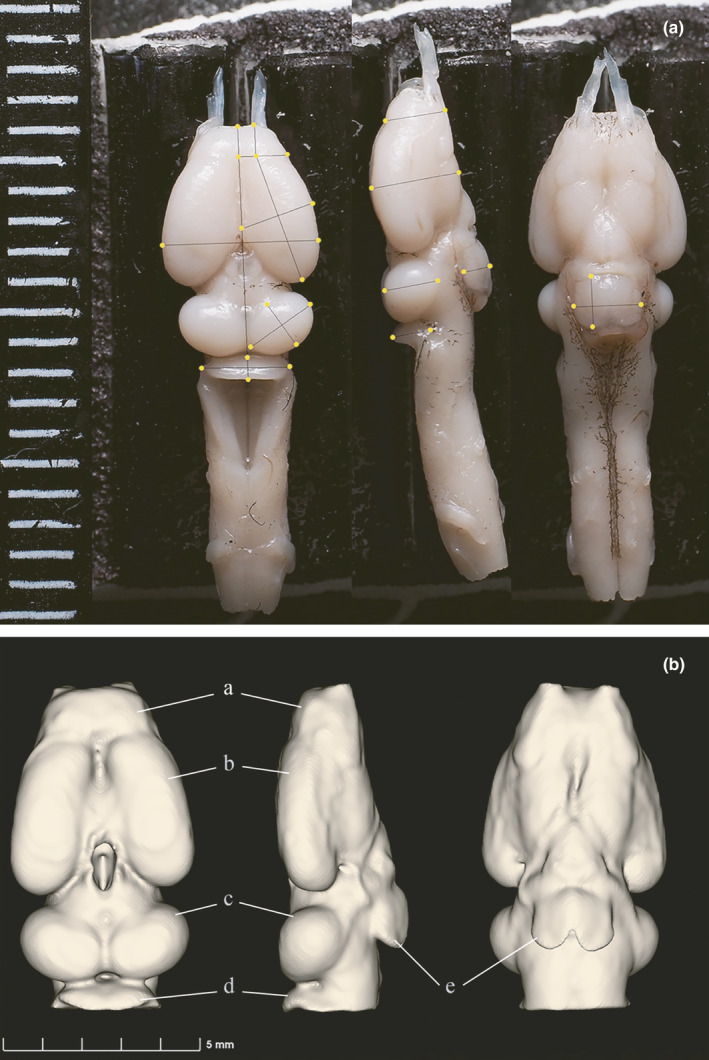
Methods of measurement and 3D modeling of the toad brain. (A) The dorsal, lateral, and ventral view of a dissected brain. The lines indicate the measured length, width, and height of brain and brain regions. (B) A completed 3D model of a toad brain that is used for volumetric estimate. Different brain regions are indicated as (a) olfactory bulb, (b) telencephalon, (c) optic tectum, (d) cerebellum, and (e) pituitary infundibulum

Length (*L*), width (*W*), and height (*H*) of brain and brain regions, including olfactory bulb (OLF), telencephalon (TEL), optic tectum (TEC), cerebellum (CER), and pituitary infundibulum (PIT), were distinguished and measured to the nearest 0.001 mm from the photographs as shown in Figure 3A (for details see Jiang et al., 2015; Zeng et al., 2016). Each of the traits was measured three times. All the measurements were conducted by the same investigator (ZY) to eliminate interobserver variability (Burns et al., 2009). The volumes of brain and brain regions were calculated using the ellipsoid model as *V* = (*L* × *H* × *W*)*π*/(6 × 1.43) (Jiang et al., 2015; Zeng et al., 2016). For OLF, TEL, and TEC, which had two symmetrical structures, only the right‐side structures were measured and the volume of the ellipsoid model was multiplied by two. Several brains or brain regions with damages were measured only on one side, so the total volumes were extrapolated assuming the brain is a symmetrical structure. Overall, a total of 268 specimens were measured for whole‐brain volume (*V*_LM_), 264 for OLF, 264 for TEL, 267 for TEC, 266 for CER, and 235 for PIT.

### 2.3 3D modeling‐based volume estimate (*V*_3D_)

Eight brain specimens per sampling site (4 females and 4 males) were randomly selected for CT scans to estimate brain volume using 3D modeling (*V*_3D_). Site 2.5 had only six samples and was excluded from this set of analysis. The brains were washed in tap water for 24 hr and then soaked in I_2_KI solution (3.00% w/v) for 45 hr (Porro & Richards, 2017). A micro‐CT machine (PerkinElmer, Quantum GX) was used to obtain the DICOM data at 70 kV, 88 μA.

A fixed threshold was used to obtain the 3D models of brains. The olfactory nerves and other parts that were not included in measurements were removed as in Figure 3B. For brains that were incompletely dyed with iodine, the models were repaired using the symmetrical half of the brain. A total of 80 brain models were constructed and measured. All of the modeling, segmentation, and volume counting were conducted in 3DSlicer (version 4.10.2; Fedorov et al., 2012).

### 2.4 Data analysis

We randomly selected 30 and 37 specimens from transect 1 and transect 2, respectively, to test repeatability of measurements, including SVL, length, width, and height of brain and brain regions. The intraclass correlation coefficient of each measurement was calculated using R package icc (Wolak et al., 2012), and all were equal to or above 0.90. Consequently, the mean values of repeated measurements were used in all downstream analyses.

To test the discrepancy between the two volumetric measurements (*V*_LM_ and *V*_3D_), the mean relative error (Bland & Altman, 1986; Tsuboi et al., 2020) was calculated as

$$\bar{r}=\frac{\sum \frac{\left|V_{\text{LM}i}‐V_{3Di}\right|}{(V_{\text{LM}i}+V_{3Di})/2}}{n}=\frac{2}{n}\sum \frac{\left|V_{\text{LM}i}‐V_{3Di}\right|}{\left(V_{\text{LM}i}+V_{3Di}\right)},$$

where *n* was the sample size, *V*_LM_ *_i_* and *V*_3D_ *_i_* were the measured volumes of the *i*th specimen. Furthermore, linear models were used to obtain a predictive equation between *V*_LM_ and *V*_3D_ using R package lme4 (Bates et al., 2014). Both *V*_LM_ and *V*_3D_ were log‐transformed, and log *V*_LM_ was set as the independent variable and log *V*_3D_ was set as the response variable.

To explore the overall patterns of brain size variation, data from the two sampling transects were first analyzed together. Linear mixed‐effects models were constructed to test the correlation between brain, brain regions, and altitude using R package lme4. The log‐transformed volume of whole brain or brain regions was set as the response variable. Sex, which was set as 0 for female and 1 for male, and altitude, which was treated as a continuous variable, were set as fixed effects. To compare the relative size, SVL (log‐transformed) was set as a covariable in model fitting when testing the whole‐brain volume, and V_LM_ was set as a covariable when exploring brain region sizes (Axelrod et al., 2018; Jiang et al., 2015). Additionally, the sampling transect was set as a random effect.

To explore the repeatability of brain size change along altitudinal gradients, data from the two transects were also analyzed separately. Linear models were used to test the correlation between brain size, brain region size, and altitude in each sampling transect using R package lme4. Similarly, the log‐transformed brain or brain region volume was set as the response variable, while sex and altitudes were set as fixed effects. SVL and V_LM_ were, respectively, set as covariables when exploring brain size and brain region size.

To meet the normality assumption of models, all SVL and volume data were log‐transformed before modeling. For all models, we ran model diagnostics to test model assumptions including normality, linearity, and homoscedasticity (Kabacoff, 2015). All of these analyses were conducted in R (vison 4.0.2; R Core Team, 2020).

## 3 RESULTS

### 3.1 Volumetric data comparison

The mean relative error between *V*_LM_ and *V*_3D_ was 0.108, which suggested a rather large difference between the two measurement methods. Based on the linear models (estimate ± *SE* = 0.743 ± 0.035, *t* = 21.200, *p* < .001, adjusted *R* ^2^ = 0.850), the predictive equation from *V*_LM_ to *V*_3D_ was log(*V*_3D_) = 0.977+0.743 × log(*V*_LM_).

### 3.2 Size of whole brain

**FIGURE 4:**
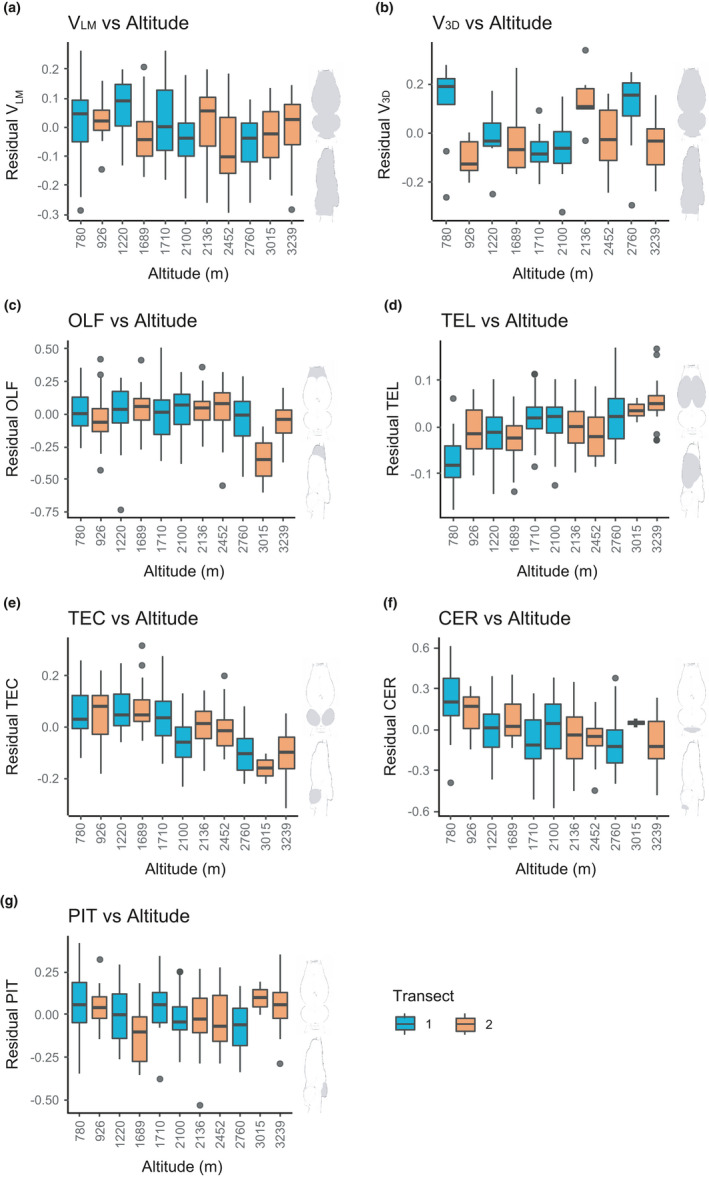
Relative size of brain and brain regions across altitudinal gradients. Each bar represents a sampling site. Blue bars are from transect 1, and orange bars are from transect 2. Plots a and b show residual brain volume to SVL, and other plots show residual brain region volume to *V*_LM_. *V*_LM_ (a), *V*_3D_ (b), TEC (e), and CER (f) decrease significantly with altitude while TEL (d) increases significantly with altitude. OLF (c) and PIT (g) show no significant trend with altitude

When data from both transects were pooled, *V*_LM_ varied from 34.603 mm^3^ to 121.934 mm^3^. The relative proportion of *V*_LM_ to SVL varied from 1.191 to 0.562, while SVL varied from 56.968 mm to 117.872 mm. The average relative volume to SVL of the lowest altitude population (site 1.1, 785 m) was 0.870, while the average value of the highest altitude population (site 2.6, 3,239 m) was 0.737. The linear mixed‐effects models revealed that the relative volume (*V*_LM_) reduced significantly with the increase in altitude (estimate ± *SE* = −0.038 ± 0.010, *t* = −3.955, *p* < .001; Table 2 and Figure 4a). When analyzed separately, transect 1 had the same reduction pattern (estimate ± *SE* = −0.064 ± 0.014, *t* = −4.495, *p* < .001; Table 3 and Figure 4a), but transect 2 did not (estimate ± *SE* = −0.013 ± 0.013, *t* = −0.973, *p* = .333; Table 3 and Figure 4a). In addition, the *V*_3D_ data revealed the same pattern when data from both transects were pooled (estimate ± *SE* = −0.035 ± 0.017, *t* = −2.062, *p* = .043; Table 2 and Figure 4b). As a whole, Asiatic toads that live at high altitudes tend to have relatively smaller brains than those at low altitudes.

**TABLE 2:** Results of linear mixed‐effects model analysis with data from the two transects pooled

Response variable	Fixed effect	Estimate	*t*‐value	*df*	*p‐*Value
Whole brain					
*V*_LM_	Sex	−0.028 ± 0.022	−1.277	267.928	.203
	**Altitude**	−0.038 ± 0.010	−3.955	267.902	**<.001**
	**SVL**	1.375 ± 0.075	18.262	264.000	**<.001**
*V*_3D_	Sex	0.042 ± 0.035	1.203	79.192	.233
	**Altitude**	−0.035 ± 0.017	−2.062	78.990	**.043**
	**SVL**	1.144 ± 0.128	8.912	79.746	**<.001**
Brain region					
OLF	Sex	0.018 ± 0.032	0.556	264.000	.579
	Altitude	−0.014 ± 0.016	−0.911	264.000	.363
	***V*_LM_**	1.216 ± 0.068	18.012	264.000	**<.001**
TEL	**Sex**	0.041 ± 0.009	4.465	264.000	**<.001**
	**Altitude**	0.039 ± 0.004	8.603	264.000	**<.001**
	***V*_LM_**	1.013 ± 0.019	53.473	264.000	**<.001**
TEC	Sex	0.009 ± 0.016	0.544	265.054	.587
	**Altitude**	−0.091 ± 0.008	−11.530	265.686	**<.001**
	***V*_LM_**	0.727 ± 0.033	22.183	265.256	**<.001**
CER	Sex	−0.000 ± 0.034	−0.011	264.300	.991
	**Altitude**	−0.128 ± 0.017	−7.431	266.000	**<.001**
	***V*_LM_**	0.817 ± 0.072	11.397	265.200	**<.001**
PIT	Sex	−0.076 ± 0.029	−2.608	233.092	.010
	Altitude	−0.019 ± 0.015	−1.263	233.834	.208
	***V*_LM_**	0.743 ± 0.063	11.783	233.263	**<.001**

Note

All brain measurements are volume. Significant relationships are in bold.

**TABLE 3:** Results of linear model analysis with data from the two transects analyzed separately

Response variable	Fixed effect	Estimate	*t*‐value	*p‐*value
Whole brain				
*V*_LM_ of transect 1	Sex	−0.070 ± 0.030	−2.304	.023
	**Altitude**	−0.064 ± 0.014	−4.495	**<.001**
	**SVL of transect 1**	1.214 ± 0.097	12.485	**<.001**
*V*_LM_ of transect 2	Sex	0.024 ± 0.031	0.770	.443
	Altitude	−0.013 ± 0.013	−0.973	.333
	**SVL of transect 2**	1.636 ± 0.121	13.542	**<.001**
Brain region				
OLF of transect 1	Sex	0.039 ± 0.050	0.773	.441
	Altitude	−0.023 ± 0.027	−0.876	.383
	***V*_LM_ of transect 1**	1.124 ± 0.103	10.868	**<.001**
OLF of transect 2	Sex	−0.008 ± 0.043	−0.183	.855
	Altitude	−0.001 ± 0.021	−0.071	.943
	***V*_LM_ of transect 2**	1.332 ± 0.092	14.548	**<.001**
TEL of transect 1	Sex	0.034 ± 0.014	2.425	.017
	**Altitude**	0.052 ± 0.008	6.800	**<.001**
	***V*_LM_ of transect 1**	1.022 ± 0.029	35.158	**<.001**
TEL of transect 2	**Sex**	0.051 ± 0.012	4.255	**<.001**
	**Altitude**	0.029 ± 0.006	5.026	**<.001**
	***V*_LM_ of transect 2**	1.010 ± 0.026	39.066	**<.001**
TEC of transect 1	Sex	−0.034 ± 0.023	−1.459	.147
	**Altitude**	−0.114 ± 0.012	−9.206	**<.001**
	***V*_LM_ of transect 1**	0.668 ± 0.048	13.990	**<.001**
TEC of transect 2	Sex	0.039 ± 0.021	1.859	.066
	**Altitude**	−0.077 ± 0.010	−7.515	**<.001**
	***V*_LM_ of transect 2**	0.759 ± 0.045	16.696	**<.001**
CER of transect 1	Sex	−0.049 ± 0.057	−0.862	.390
	**Altitude**	−0.146 ± 0.030	−4.800	**<.001**
	***V*_LM_ of transect 1**	0.832 ± 0.117	7.092	**<.001**
CER of transect 2	Sex	0.042 ± 0.039	1.069	.287
	**Altitude**	−0.116 ± 0.019	−6.092	**<.001**
	***V*_LM_ of transect 2**	0.764 ± 0.085	9.026	**<.001**
PIT of transect 1	Sex	−0.057 ± 0.044	−1.290	.200
	**Altitude**	−0.050 ± 0.023	−2.151	**.034**
	***V*_LM_ of transect 1**	0.791 ± 0.092	8.564	**<.001**
PIT of transect 2	**Sex**	−0.097 ± 0.039	−2.478	**.015**
	Altitude	0.005 ± 0.019	0.270	.788
	***V*_LM_ of transect 2**	0.689 ± 0.088	7.840	**<.001**

Note

All brain measurements are volume. Significant relationships are in bold.

### 3.3 Size of brain regions

When data from the two transects were analyzed together, the largest brain region was TEL. Its average relative proportion (to whole‐brain volume, *V*_LM_) in the lowest altitude population was 0.461, while the value in highest altitude population was 0.532. However, the average proportions of TEC and CER in the lowest altitude population were 0.133 and 0.025, and the values in the highest altitude population were 0.100 and 0.016. A significant reduction pattern was detected for TEC (estimate ± *SE* = −0.091 ± 0.008, *t* = −11.530, *p* < .001; Table 2 and Figure 4e) and CER (estimate ± *SE* = −0.128 ± 0.017, *t* = −7.431, *p* < .001; Table 2 and Figure 4f). There was no trend of reduction for OLF and PIT (Table 2 and Figure 4c,g). However, TEL significantly enlarged along with increase in altitudes (estimate ± *SE* = 0.039 ± 0.004, *t* = 8.603, *p* < .001; Table 2 and Figure 4d). Clearly, different brain regions have different patterns of size change along the altitudinal gradients.

When data from the two transects were analyzed separately, TEL, TEC, and CER had the same pattern between the two transects. The relative size of TEL enlarged with increase in altitudes in both transect 1 (estimate ± *SE* = 0.052 ± 0.008, *t* = 6.800, *p* < .001; Table 3 and Figure 4d) and transect 2 (estimate ± *SE* = 0.029 ± 0.006, *t* = 5.026, *p* < .001; Table 3 and Figure 4d). Meanwhile, the relative sizes of TEC and CER reduced with increase in altitudes in both transects (TEC: transect 1, estimate ± *SE* = −0.114 ± 0.012, *t* = −9.206, *p* < .001; transect 2, estimate ± *SE* = −0.077 ± 0.010, *t* = −7.515, *p* < .001; CER: transect 1, estimate ± *SE* = −0.146 ± 0.030, *t* = −4.800, *p* < .001; transect 2, estimate ± *SE* = −0.116 ± 0.019, *t* = −6.092, *p* < .001; Table 3 and Figure 4). In addition, the PIT of transect 1 had a marginally significant reduction pattern (estimate ± *SE* = −0.050 ± 0.023, *t* = −2.151, *p* = .034; Table 3 and Figure 4g).

### 3.4 Sexual dimorphism in brain size

For males, the average relative proportions of TEL and PIT were 0.503 and 0.026, while for females, they were 0.497 and 0.03. There was a significant sexual dimorphism in TEL (estimate ± *SE* = 0.041 ± 0.009, *t* = 4.465, *p* < .001; Table 2) and PIT (estimate ± *SE* = −0.076 ± 0.029, *t* = −2.608, *p* = .010; Table 2). Female toads have smaller telencephalon and larger pituitary infundibulum than males at all sampling altitudes. Other brain regions showed no significant sexual dimorphism.

## 4 DISCUSSION

The relative brain size of Asiatic toads reduces along with the increase in altitudes. Different brain regions do not change their relative sizes in a concerted fashion; while the sizes of optic tectum and cerebellum decease along with altitudes, telencephalon increases its size at high altitudes.

### 4.1 Expensive brain and cognitive buffer?

Our data support EBH. There is a trend of relative brain size reduction along with the increase of altitudes (Table 2 and Figure 4), and this is consistent with the predictions of EBH. At high altitudes, the toads face multiple environmental stressors, including hypoxia, low temperature, and dehydration (Bouverot, 1985). Hypoxia and low ambient temperature affect energy supply, metabolism, and life‐history traits in anuran species (Muir et al., 2014; Yu et al., 2014). Furthermore, a combination of dehydration and cold temperature leads to shorter active periods in both day–night cycle and seasonal cycle, which also limits the energy supply. Organisms at high altitudes have to balance the limited energy to keep all the functional organs at a necessary level (Kotrschal et al., 2016, 2019). The reduced size of the brain, an energetically expensive organ, at high altitudes is likely adaptive. It should be noted that the significant reduction pattern was observed only in transect 1 and when data are pooled. It is statistically significant, but the pattern itself is relatively weak (Figure 4). This is likely associated with the mosaic evolution of brain regions (see below).

Our data do not reject CBH and are partially consistent with the predictions of CBH. Environmental variability often requires high cognitive capacity and therefore favors larger brains (Sayol et al., 2016). Nevertheless, whether environmental challenges lead to a large brain depends on both the cognitive benefits and physical environment (Fong et al., 2019); only when the brain or a given region acquires the performance or fitness that offsets the increased energy demand, the brain or brain region may increase its size (Axelrod et al., 2018; De Meester et al., 2019; Safi et al., 2005). In turn, extra investment for a specific brain region could reflect the increased demands in response to biotic or abiotic challenges (Puga et al., 2018). Although the whole brain reduces in size, the telencephalon increases its size along the altitudinal gradients in both transects. This is consistent with the predictions of CBH. The telencephalon is associated with motivational state, learning, and spatial memory (Kardong, 2012). Toads at high altitudes likely face additional cognitive challenges, which demand an enlarged telencephalon to cope with it. Previous studies of guppies (*Poecilia reticulata*) also concluded a functional trade‐off between increased cognitive ability and reproductive performance (Kotrschal et al., 2013).

The CBH and EBH are not mutually exclusive (Heldstab et al., 2019; van Woerden et al., 2012), and the size variations of vertebrate brains are probably the results of a balance between energy allocation and cognitive benefits (Kotrschal, Corral‐Lopez, et al., 2015; Kotrschal et al., 2013; Lázaro et al., 2018). While the EBH emphasizes on energetic constraints (LemaîTre et al., 2009), CBH focuses on cognitive benefits.

### 4.2 Concerted or mosaic evolution?

Clearly, the brain regions of Asiatic toads do not evolve in a concerted fashion. The optic tectum and cerebellum decrease in relative size with increased altitudes, whereas telencephalon increases in size, and the olfactory bulb and infundibulum do not have a consistent trend along altitudes. Therefore, the brain variation of Asiatic toads along altitudinal gradients supports the functional constraints hypothesis. Selection pressure on different brain regions may vary, depending on ecological differences in the environments, which causes the brain regions to evolve independently (Gonzalez‐Voyer & Kolm, 2010; Huang et al., 2020; Liao et al., 2015; Safi & Dechmann, 2005). In the case of Asiatic toads at high altitudes, they are sampled during the breeding season, and finding breeding sites (ponds), which are scarce at high altitudes, is likely more important than foraging or antipredation. As a consequence, investment for optic tectum and cerebellum is reduced and investment for telencephalon is increased. Previous studies examining other ecological differences, including habitat complexity (Safi & Dechmann, 2005), environmental matching locomotor mode (Macrì et al., 2019), and seasonality (Lázaro et al., 2018), also reached similar conclusions.

Whether the brain is a collection of independently varying structures or a unitary coordinated processing structure is a core debate of brain evolution (Yopak et al., 2010). The DCH and FCH are not mutually exclusive (Montgomery et al., 2016), and distinguishing one from the other can be difficult. Although different parts may evolve at different rates, some degree of brain allometry is often retained and most brain part sizes are highly predictable from the whole‐brain size (Tsuboi et al., 2018; Yopak et al., 2010). In the case of Asiatic toads, the relative sizes of the optic tectum and cerebellum followed the reduction trend of the whole brain. Additionally, moderate selective pressures on all brain regions may make it appear to be concerted. Furthermore, different regions have different potentials in evolvability or plasticity, which constrains the size change in regions (Lázaro et al., 2018). Conditions in early life also have a larger effect on brain region sizes than experience in later life (Burns et al., 2009). All these factors may constraint the independence of brain regions. Further genetic and developmental examinations will be helpful to clarify mechanisms behind coordination of brain regions (Kotrschal et al., 2017).

The repeated patterns between the two sampling transects reinforce the general patterns for brain structure variation along altitudinal gradients. The two transects are from two different mountain ridges and variation patterns along each transect likely evolved independently. Furthermore, we have controlled the geographic distance within each transect to less than 85 km, thus reduced potential impacts of other confounding factors. Therefore, the observed patterns are likely caused by altitudinal gradient and associated environmental factors. It should be noted that the reduction trend of the whole brain is not repeated between the two transects; the weak pattern is likely a consequence of different brain regions having opposite trends.

### 4.3 Brain size variation in amphibians and other vertebrates

Two early studies examined amphibian brain size variation along altitude. Mai et al. (2017) compared brain size variation in the Asian Grass Frog (*Fejervarya limnocharis*) across different latitudes and altitudes. Altitude did not affect relative brain size of this species, but there was a positive correlation between cerebellum and altitude (Mai et al., 2017). This is different from our results. Several factors may have caused these differences. First, the altitudinal difference in the Grass Frog is small (0–900 m) and sampling sites scatter across a large region (Mai et al., 2017). This likely introduces large random effects. Second, the observed patterns could be species specific. Another similar study is an intraspecific comparison of the Andrew's Toad (*Bufo andrewsi*; Jiang et al., 2015). They detected positive correlations between the whole brain (both absolute and relative size), olfactory bulb, optic tectum, and the length of active season. The latter is determined by altitude and latitude. The finding is similar to ours, even though we did not find a relationship between altitude and olfactory bulb.

Several potential causes for the morphological variations of the brain have been examined but the results so far are mixed. Seasonality, which includes temperature and some other factors, is negatively associated with the relative size of the brain and optic tectum in some frogs (Luo et al., 2017), but does not affect the Guenther's frog (*Hylarana guentheri*; Gu et al., 2017). Seasonality also has a positive impact on avian brains (Sayol et al., 2016), but has a negative impact on other endotherms (van Woerden et al., 2010, 2012). Severe seasonality is one of the climatic features of high altitudes. In the case of Asiatic Toads, size changes in the whole brain, cerebellum, and optic tectum are negatively correlated with seasonality, but the telencephalon is positively correlated with it. Habitat complexity or type, which is more general than seasonality, is another potential cause. It does not influence brain morphology in reptiles (De Meester et al., 2019) or mammals (Towe & Mann, 1995). However, brain size or structure is associated with habitat complexity or type in primates (Powell et al., 2017), fishes (Axelrod et al., 2018; Kotrschal et al., 2017), and anurans (Liao et al., 2015). Currently, there is a general lack of consensus in regard to the causes of variations, and much research is needed in this area.

### 4.4 Limitations and future direction

There are several limitations of our study. We only examined brains in the breeding season. Although our sampling strategy should remove seasonal change from our comparison, patterns recovered from our data may not represent brain size variations in other annual seasons. Seasonal plasticity of bran size and structure is well documented across different taxa (Axelrod et al., 2018; Puga et al., 2018; Sampedro et al., 2008; Stahn et al., 2019). In addition, we did not include age as a covariate. Relative brain volume is evidently associated with age (Jiang et al., 2015). Adult Asiatic toads are mostly 2–5 years old but reliably aging toads are difficult. Lastly, this is a correlational study; the explanation of the statistic models is not sufficient. To establish causation, other energetic organs should be taken into consideration and experiments of functional verification are needed.

Computed tomography has recently become popular in research of neuroanatomy (Kotrschal et al., 2017; Macrì et al., 2019; Smith et al., 2016), in parallel to the more traditional dissected measurements (Bauchot et al., 1977; Harvey et al., 1980; Zeng et al., 2016). We compared a CT scan 3D model‐based volumetric estimate with a dissected linear measurement‐based estimate, and detected a large discrepancy between them (mean relative error = 0.108). The small sizes of these brains and their irregular shapes likely contributed to measurement errors, and we also have a relatively small sample size for the 3D model. With a large discrepancy, the predictive equation may not be very useful. With the present data, it is difficult to conclude which method is better, and clearly, more testing and refinement of these measurement methods are needed.

The altitude gradient appears to be a promising system for studying brain size variation. More exploration conducted along environmental gradients is required to clarify the complicated mechanisms behind the adaptive evolution of the brain (Albert et al., 2010).

## 5 CONCLUSION

We explored size variation of the brain and brain regions along altitudinal gradients. The relative size of the whole brain, optic tectum, and cerebellum decreases with the increase of altitude, while the relative size of the telencephalon increases with the altitude. Our results support both the expensive brain hypothesis and the functional constraints hypothesis; however, the change in telencephalon size supports the cognitive buffer hypothesis. Size variation of the brain is likely the result of a trade‐off between energetic expenditure and cognitive capacity.

## CONFLICT OF INTEREST

None declared.

## AUTHOR CONTRIBUTION

**Zhongyi Yao:** Conceptualization (equal); Investigation (lead); Writing‐original draft (lead); Writing‐review & editing (equal). **Yin Qi:** Data curation (equal); Formal analysis (equal); Software (equal); Writing‐original draft (supporting); Writing‐review & editing (equal). **Bisong Yue:** Conceptualization (equal); Supervision (equal); Writing‐original draft (supporting); Writing‐review & editing (supporting). **Jinzhong Fu:** Conceptualization (equal); Funding acquisition (lead); Project administration (lead); Supervision (lead); Writing‐original draft (equal); Writing‐review & editing (equal).

## Data Availability

All the data generated for this study have been made available on Dryad Digital Repository. https://doi.org/10.5061/dryad.mcvdncjzc.
